# Potential protective effect of 3,3′-methylenebis(1-ethyl-4-hydroxyquinolin-2(1*H*)-one) against bleomycin-induced lung injury in male albino rat via modulation of Nrf2 pathway: biochemical, histological, and immunohistochemical study

**DOI:** 10.1007/s00210-022-02324-1

**Published:** 2022-12-08

**Authors:** Sara Mohamed Naguib Abdel Hafez, Entesar Ali Saber, Neven Makram Aziz, Maha Yehia Kamel, Ashraf A. Aly, El-Shimaa M. N. Abdelhafez, Manar Fouli Gaber Ibrahim

**Affiliations:** 1 Department of Histology and Cell Biology, Faculty of Medicine, Minia University, Minia, 61111 Egypt; 2 Delegated to Deraya University, New Minia City, Minia Egypt; 3 Department of Medical Physiology, Faculty of Medicine, Minia University, Minia, Egypt; 4 Department of Pharmacology, Faculty of Medicine, Minia University, Minia, Egypt; 5 Department of Chemistry, Faculty of Science, Minia University, Minia, Egypt; 6 Department of Medicinal Chemistry, Faculty of Pharmacy, Minia University, Minia, Egypt

**Keywords:** 3,3′-Methylenebis, Bleomycin, Lung injury, Nrf2 pathway

## Abstract

Acute lung injury is a serious condition accounting for the majority of acute respiratory failure. Bleomycin (BLM) is an antibiotic that was first described as a chemotherapeutic agent. 3,3′-methylenebis(1-ethyl-4-hydroxyquinolin-2(1*H*)-one) was reported to have anti-inflammatory, anti-apoptotic, and anti-oxidative properties. The current work aimed to assess the possible protective effects and the mechanism of protection of 3,3′-methylenebis-(1-ethyl-4-hydroxyquinolin-2(1*H*)-one) on BLM-induced lung injury in addition to the effect and underlying mechanisms of nuclear factor-erythroid-related factor 2 pathway against this injury. Rats were equally divided into four groups: control group, BLM group, 1-ethyl-4-hydroxyquinolin-2(1*H*)-one-treated group, and BLM with 1-ethyl-4-hydroxyquinolin-2(1*H*)-one-treated group. At the end of the work, the blood samples were proceeded for biochemical study. Lung specimens were obtained for biochemical, histological, and immunohistochemical study. The results exhibited a significant increase in both malondialdehyde and tumor necrotic factor-α with a significant decrease in glutathione, superoxide dismutase, IL 10, surfactant protein A, and nuclear factor erythroid 2-related factor 2 in BLM group. The lung histological results showed various morphological changes in the form of disturbed architecture, inflammatory cell infiltration, and intraluminal debris. This group also displayed a significant increase in the mean surface area fraction of anti-cleaved caspase 3, while group IV exhibited amelioration in the previously mentioned parameters and histological alternations that were induced by BLM. It could be concluded that 3,3′-methylenebis(1-ethyl-4-hydroxyquinolin-2(1*H*)-one) has anti-oxidative, anti-inflammatory, and anti-apoptotic protective effects against BLM-induced lung injury.

## Introduction

Acute lung injury (ALI) and acute respiratory distress syndrome (ARDS) (Zhao et al. 2022) are characterized by lung vascular permeability, pulmonary edema, massive alveolar damage, and recruitment of numerous inflammatory cells to the lungs. Loss of control over the migration of inflammatory cell infiltrations toward the inflamed lung tissue shares in the pathology of life-threatening ALI and its severe form; ARDS (Pouzol et al. 2021).

Bleomycin (BLM)-induced lung injury is represented in 40–45% of the patients receiving BLM and has a mortality rate of 1–3% (Necchi et al. 2017). Multiple forms of interstitial lung disease had been reported. BLM-induced lung injury is similar to other drug-induced pneumonitis cases. Steroids induced short-term symptomatic relief in about 50–70% of patients, but symptoms recurred during steroid reduction therapy. Thus, patients with BLM-induced lung injury with diffuse alveolar damage could not be treated with steroids alone (Ro et al. 2020).

Idiopathic pulmonary fibrosis (IPF) is known as a progressive, life-threatening interstitial lung tissue disease with about a median survival of 3–5 years (Xue et al. 2020). But, as the unclear cause and problems with inaccurate diagnosis, until now, only two drugs were approved by Food and Drug Administration for the treatment of IPF and their responses to the results are limited. The discovery of quinolone-based drugs has attracted more attention from all researchers due to their diverse spectrum of biological activities (Özdemir 2016) as some compounds had a potential for protection and properties modification of natural beside synthetic materials e.g. antifungal agents, antiviral effects, and anti-microbial activity (Michael 2002; Paeshuyse et al. 2008). It was reported on quinolones’s anti-cancer activity as they were able to suppress cancer cell proliferation (Elbastawesy et al. 2019, 2020; Mohamed and Ramadan 2020). However, other 2-quinolones were shown as anti-apoptosis activity (Aly et al. 2020).

Nuclear factor-erythroid-related factor 2 (Nrf2) controls the expression of most antioxidant genes. Bach 1 is a competitive inhibitor of Nrf2 through the competitive interference and suppression of Nrf2 interaction and antioxidant response element. This negatively controls antioxidant genes as heme oxygenase 1 (HO-1) and glutathione peroxidase 1 (Gpx1). Nrf2 and its antioxidant elements downstream play a critical role in the pathology of lung injury. Nrf2 agonist BLM-induced attenuated lung injury *via* oxide level in lung tissue (Liu et al. 2019). A previous report demonestrated that Bach 1 knockdown controlled the progress of BLM-induced pulmonary fibrosis by controlling expressions of both Nrf2 and its downstream antioxidant elements as Ho-1 and Gpx1, so providing new insights into the role of Bach 1/Nrf2 in the control of oxidative stress involved in pathogenesis. in lung injury (Liu et al. 2017).

Based on the above findings, this paper aimed to assess the possible protective effects and the mechanism of protection of 3,3′-methylenebis-(1-ethyl-4-hydroxyquinolin-2(1*H*)-one) on BLM-induced lung injury in addition to the effect and underlying mechanisms of nuclear factor-erythroid-related factor 2 pathway against this injury.

## Material and methods

### Ethical approval

This study was approved by Ethics Committee “FMREC” Faculty of Medicine, Minia University, Minia, Egypt, regarding the source of the rats, inclusion criteria, exclusion criteria, caging, comfort, health status, and the detailed experimental design and procedures. Approval No. 58:8/2021.

### Animals

In the current study, 40 adult male albino rats weighing about 200–225 gm, aged 4–6 weeks were used. Rats were purchased from the animal house, Faculty of Medicine, Minia University, and all rats were housed in a clean air-conditioned room. Free access to water and a commercial diet to the rats were allowed (Nile Company, Egypt). Two weeks for acclimatization at room temperature were allowed with 12 h dark/12 h light cycles.

### Drug protocol

Chemicals were purchased from Aldrich, Alfa Aesar, Across Organics, and El-Nasr Pharmaceutical Chemical Companies and used without further purification. The reaction was monitored using pre-coated Thin Layer Chromatography plates (Kiesslgel 60, 254 Merck), and the spots were assessed by exposure to a UV lamp at 254 nm. Analytical grade chemicals and solvents were used, and the reactions were observed by a pre-coated Merck silica gel 60 PF_25__4_ aluminum sheet. Melting points were monitored on a Stewart electrothermal melting point apparatus in degrees Celsius (°C) and were not corrected. When equal amounts of 4-hydroxy-2 (1*H*)-quinolones and Et_3_N were added and gently heated in the oil track at 70–80 °C with DMF for 10 h, the resulting yellowish-orange color of the solution gradually turned brown and 3,3′-methylenebis (substituted 4-hydroxyquinolin-2(1*H*)-one (**1**) was precipitated in 70% yield. The structural elucidation and purity confirmation of compound **1** were obtained utilizing different spectroscopic techniques such as NMR, IR, and mass spectra. X-ray structure analyses were recorded in the reported literature (Aly et al. 2021) (see the scheme of synthesis in the supplementary file).

#### Bleomycin sulfate (available as 15 U Bleocip injection, Cipla)

At 100 mU, it was done in 30 μl of sterile saline and then slowly infused by the catheter into the trachea.

#### Rat model of bleomycin-induced lung injury

Rats were carefully anesthetized with a mixture of ketamine (80 mg/kg body weight) and xylazine (5 mg/kg body weight) and placed on an intubation holder facing upwards at an angle of about 45° using a flexible suture carefully placed under the animal.

#### Front incisors

Rat tongues were gently pulled out with forceps and the trachea was intubated using a sterile 22-G plastic tube intravenously catheters.

### Experiment protocol

Rats were divided into equally four groups of 8 rats each:

Group I serving as a control group, is subdivided into:

- Group Ia: control animals were injected intra-tracheally with one dose of 0.1 ml sterile saline (solvent of BLM).
- Group Ib: animals were injected intra-peritoneal with one dose of 0.1 ml DEXA (solvent of 3,3′-methylenebis).

Group II (BLM group): Each animal was injected intra-tracheally with one dose of (7.5 IU/kg) bleomycin dissolved in 0.1 ml sterile saline and sacrificed after 48 h of injection; modified (Zargar et al. 2017).

Group III (3,3′-methylenebis-treated group): Each animal was injected intra-peritoneal with one dose of 3,3′-methylenebis (100 mg /kg) dissolved in 0.1 ml DEXA (Osheroff et al. 2013).

Group IV (BLM with 3,3′-methylenebis-treated group): Each rat was injected intra-peritoneal with one dose of 3,3′-methylenebis (100 mg /kg) dissolved in 0.1 ml DEXA. After 24 h, rats received a single dose of (7.5 IU/kg) bleomycin dissolved in 0.1 ml sterile saline. Then, the animals were sacrificed after 48 h of injection.

The two lungs were rapidly taken out, washed with ice-cold saline, and then split into 3 parts. The first lung part (10% w/v) was homogenized by using a Teflon tissue homogenizer (Omni International Inc., Kennesaw, GA, USA) in phosphate-buffered saline (PBS), after that for about 10 min by using a centrifuge at about 3000 rpm the clear homogenates were obtained and frozen at − 80° C for subsequent analysis of the oxidative stress parameters. The second part was kept frozen at − 80° C for gene and protein expression analysis. Finally, the third part was obtained for electron and light microscopic examination.

### Biochemical analyses

At the end of the work, the rats were decapitated after overnight fasting, and the blood samples were collected from the left jugular vein at room temperature in a tube containing 0.5% heparin as the anticoagulant then centrifuged in a cooling (Hettich) centrifuge at about 3000 rpm for 15 min. After the obtained clear plasma was obtained, it was stored at about−80 °C until used for Surfactant protein A (SP-A) level by using Rat’s SP-A ELISA kit (GENTAUR, USA) regarding the manufacturer’s instructions.

### Analysis of lung homogenates

The right lung tissue was rapidly removed from each animal and then washed with ice-cold saline. Then was immediately submerged in liquid nitrogen then stored at − 80 °C for the biochemical analysis. After then, the lung was homogenized in cold potassium phosphate buffer (0.05 M, pH 7.4). The tissue homogenates were centrifuged at 5000 rpm (revolutions/rotor) for about 10 min at 4 °C. The resulting supernatant was used for the measurement of:

- Malondialdehyde (MDA) content regarding the method of (Ohkawa et al. 1979).
- Antioxidant activity was measured by detecting both reduced glutathione (GSH), and superoxide dismutase (SOD). A colorimetric assay to measure GSH concentration was carried out, and the level of GSH was detected at about 412 nm by using a spectrophotometer. The findings were expressed as µ0mol/g tissue protein (Vardi et al. 2008). Xanthine/xanthine oxidase assay was carried out to assess SOD (Superoxide Dismutase Assay Kit, Item No. 706002; Cayman Chemical Company, Ann Arbor, USA) by detecting the amount of reduced nitro blue tetrazolium (NBT) with one unit of SOD, which is detected as the amount of protein that inhibits the rate of NBT reduction to 50%. SOD was estimated as units/mg of protein tissue (Vardi et al. 2008).
- Tumor necrosis factor-alpha (TNF-α) and interleukin 10 (IL-10) levels via using Rat’s ELISA kit (Lab Vision Corporation, USA) regarding the manufacturer’s instructions.
- Nuclear factor erythroid 2-related factor 2 (Nrf2) concentration by using the Nrf2 ELISA kit (Fine Test, China) regarding to the manufacturer’s instructions.

### Fractional analysis of cells in BAL fluid

At the end of the current study, after using atropine and hydroxyzine, a bronchoscope was inserted under local anesthesia using lidocaine. The purpose of this lavage was to evaluate its cellular morphologic features. The lavage was carried out by using a 30 ml of sterile 0.9% saline that was assigned as “whole airway” lavage. The tissue was stained by Giemsa stain. The differential cell types were examined under a light microscope (Taniuchi et al. 2009).

### Histological examination

#### Light microscopic study

Left lung tissue samples from all rats were carefully dissected. Some specimens were immersed immediately in 10% buffered formalin (pH 7.2) for 48 h then processed and embedded in paraffin wax to prepare sections of 5 μm thickness stained with hematoxylin and eosin (H&E) and immunohistochemical study. Other specimens were post-fixed in 1% osmium tetroxide, then were processed and prepared as semi-thin sections stained by 1% toluidine blue. The remaining specimens were processed into ultrathin Sects. (80–90 nm) were stained with uranyl acetate 5% for 15 min and lead citrate for 8 min (Suvarna et al. 2018).

##### Immunohistochemical staining using anti-cleaved caspase

The positive control of *anti*-cleaved caspase was the rat’s tonsil, showing positive cytoplasmic and/ or nuclear deposits. Negative control specimen of the lungs was processed in the same way but omitting the step of the primary antibody.

Immunohistochemistry was performed on 5 μm lung sections for the detection of anti-cleaved caspase 3 antibody. Briefly, the sections were deparaffinized then rehydrated and then pretreated with 0.01% hydrogen peroxide to block any endogenous peroxidase activity. Sections were embedded in 0.01 M citrate buffer (pH 6) for 10 min for antigenic site unmasking followed by antigen retrieval in EDTA buffer in the microwave for 20 min. Thereafter, sections were incubated in *anti*-caspase antibodies for 1 h at room temperature. *Anti*-Caspase 3 antibody; active (cleaved) form (a marker for apoptosis) was used. It is a polyclonal rabbit antibody; Catalog Number (AB3623); used at a concentration 1:10 dilution from Merck KGaA, Darmstadt, Germany. Then, the slides were incubated in the avidin–biotin complex for 1 h. Sections were washed and incubated in peroxidase substrate (DAB) solution for 10 min. Finally, the sections were dehydrated in absolute alcohol, cleared by xylene, and mounted (Buchwalow and Böcker 2010).

#### Ultrastructural study

Smaller tissue pieces (1 × 1 mm) of all groups were immediately fixed at 4° C for 18–24 h in 3% glutaraldehyde-formaldehyde, phosphate buffer rinsing, followed by 1% osmium tetroxide post-fixation. In a sequence of alcohols, the specimens were then dehydrated, washed in propylene oxide, and eventually embedded in Epon epoxy resin. After that, an ultra-microtome trimmed the blocks, sectioning them with glass knives. Semi-thin Sects. (1 mm) had been treated with a stain; toluidine blue then examined by a light microscope to detect the correct area for the ultrathin parts. The same ultra-microtome was used also to cut ultrathin sections of about 70–90 nm, and then stained with both uranyl acetate and lead citrate stain. Joel CX 100 transmission electron microscope which is adapted to a voltage of about 60 kV examined the sections (Bozzola and Russell 1999).

### Image capture

In Histology and Cell Biology Department, both H&E, immune-stained, and semi-thin slides were studied by using an Olympus microscope (BX51, Japan). High-resolution color digital camera (Olympus, Japan) which was mounted to the microscope adapted to a computer was used to digitally capture the slides. The ultrathin sections were examined by using a transmission electron microscope (SEO-Russia), JEOL (JSM 1400 plus) in Science Faculty, Alexandria, Egypt.

### Quantitative morphometric study

The data were obtained by using the “Top view” image analyzer computer system (China). The image analyzer formed of both colored video camera, colored monitor, and hard disc of IBM personal computer adapted to the microscope and controlled by software named “Top view.” This morphometric study was carried out in Histology and Cell Biology Department, Faculty of Medicine, Minia University. To convert the measurement units (pixels) produced by the image analyzer program into actual micrometer units, the image analyzer was automatically first calibrated. Five sections from each group were used. The following parameters were measured:

- The mean area fraction for immune-positive stained cells × 400 in 10 non-overlapping fields from each section.
- The mean number of pneumocyte type II: counting the pneumocyte type II (cuboidal in shape with central rounded nuclei), the magnification used for this parameter was × 1000 and was performed in 10 non-overlapping fields from each section.
- The degree of inflammation and destruction was scored for each group (Gokakin et al. 2013) (Table 1) by using 10 non-overlapping fields from each slide from each group. We calculated the mean score in each of the variables. The total histopathological score was derived from the sum of the mean scores of the variables. The mean value of each animal group was used for statistical analysis (Table 2).

**Table 1:** Scoring of inflammation and destruction

Pathological lesion	Score
Edema	1
Hyperemia	1
Thickness in interalveolar septum	2
Mononuclear cell infiltration	2
Loss of alveolar epithelium	3
Hemorrhage	3
Total	12

**Table 2:** Energy scores for the complexes formed by the tested compound 1 and the reference 1 (N1-formyl-tryptophan) in the active site of SARS-CoV-2 enzyme (PDB:6LU7) (*n* = 8)

Compounds	S score	Residue	Type of interaction	ΔG (Kcal/mole)	Length (Å)
1	− 6.53	GLN189 GLY143 HIS41 MET49 GLU166	H-donor H-acceptor H-Pi Pi-H Pi-H	− 0.7 − 0.9 − 1.3 − 0.7 − 0.6	2.99 3.32 3.94 4.03 4.52
2	− 4.99	MET165 GLN189	Pi-H Pi-H	− 0.6 − 1.0	3.72 3.73

### Statistical methods

Statistical analysis for the biochemical and morphometric results was performed. Comparisons between groups were done using ANOVA (analysis of variance) and then post hoc Tukey test (SPSS package version 22, SPSS Inc., Chicago, USA). Results were recorded as mean and standard deviation (SD) and considered statistically significant when *P* < 0.05.

## Results

### Assessment of the oxidative status and inflammatory markers

The data of the current work in Table 3 demonstrated that the tissue MDA and TNFα in group II showed the highest level and tissue nuclear factor erythroid 2-related factor 2 (Nrf2) and IL 10 showed the lowest level if compared to all groups. However, group IV displayed a significant amelioration in the MDA, Nrf2, and TNFα levels if compared to group II but still significantly differ from the other groups, while GSH and SOD tissue levels were significantly decreased in group II if compared with the control group. Group IV showed a significant improvement in these parameters if compared to group II.

**Table 3:** Pulmonary oxidative, anti-oxidative, and inflammatory markers in the different experimental groups

Groups	Control groups		Group II	Group III	Group IV
	Ia	Ib			
MDA (nmol/gtissue)	99.53 ± 6.82	112.91 ± 7.32	195.7 ± 5.24^**ab**^	114.5 ± 6.31^**c**^	138.6 ± 4.71^abcd^
Nrf2 (pg/mg pro)	5.84 ± 0.32	6.01 ± 0.21	2.94 ± 0.01^**ab**^	9.06 ± 0.36^**abc**^	7.15 ± 0.41^abcd^
SOD (units/mg tissue protein)	4.633 ± 0.9522	4.767 ± 0.4967	2.100 ± 0.2366^**ab**^	4.750 ± 0.6834^**abc**^	3.483 ± 0.4215^abcd^
GSH (µmol/g tissue protein)	6.083 ± 0.5879	5.983 ± 0.4665	2.450 ± 0.4278^**ab**^	6.200 ± 0.1414^**abc**^	5.083 ± 0.2137^abcd^
IL 10 pg/mg tissue protein)	75.00 ± 3.162	72.67 ± 4.844	32.67 ± 1.751^**ab**^	76.00 ± 1.414^**abc**^	56.17 ± 2.041^abcd^
TNF-α (pg/mg tissue protein)	30.21 ± 1.67	32.72 ± 1.74	61.42 ± 1.62^**ab**^	34.36 ± 1.83^**c**^	42.63 ± 1.71 ^abcd^

Results represent the mean ± S.D. ^a^Significant difference from control group Ia, ^b^significant difference from control group Ib, ^c^significant difference from group II; ^d^significant difference from group III, *P* < 0.05. *MDA*, malondialdehyde; *Nrf2*, nuclear factor erythroid 2-related factor 2; *TNF-α*, tumor necrosis factor-alpha (*n* = 8)

### Assessment of the lung injury marker (surfactant protein A “SP-A”)

The result of the current study exhibited that the plasma level of SP-A in group III showed an insignificant difference when compared with control groups (Ia & Ib). However, the plasma level of SP-A was highest in group II among all experimental groups. In addition, group IV pretreated with 3,3′-methylenebis showed a significantly lower plasma level of SP-A if compared to group II, but it remained significantly higher than that of groups Ia, Ib, and III (Table 4).

**Table 4:** Plasma levels of surfactant protein A in the different experimental groups

Groups	Control groups		Group II	Group III	Group IV
Parameters	Ia	Ib			
SP-A	5.91 ± 0.29	5.88 ± 0.24	3.51 ± 0.21^ab^	5.80 ± 0.23^c^	4.55 ± 0.33 ^abcd^

Results represent the mean ± S.D. ^a^Significant difference from control group Ia, ^b^significant difference from control group Ib, ^c^significant difference from group II; ^d^significant difference from group III, *P* < 0.05. *SP-A*, surfactant protein A (*n* = 8)

### Light microscopic examination (H&E results)

**Fig. 1:**
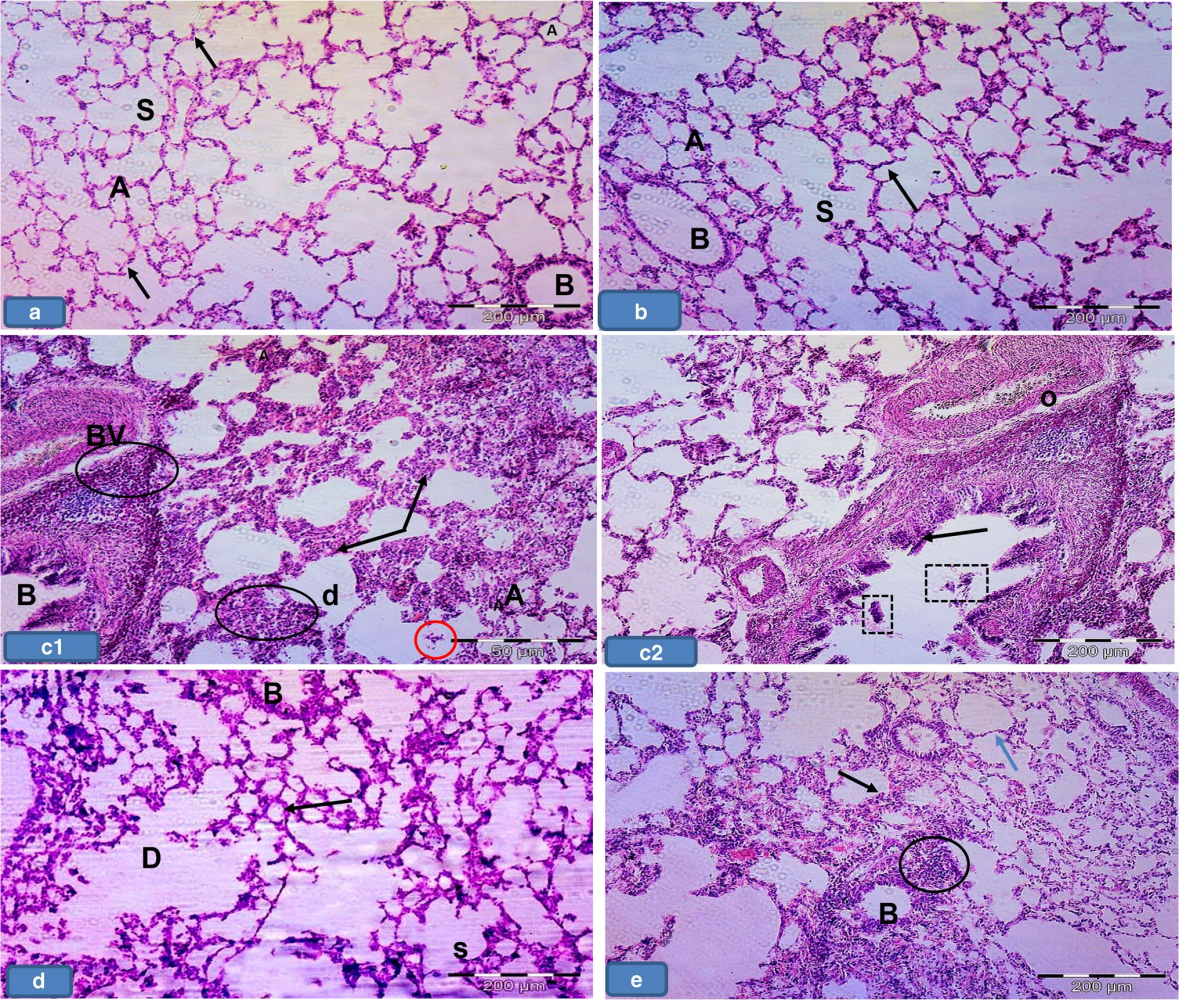
Representative photomicrographs of lung sections of adult rats: **a**, **b**, and **d** Group Ia, Ib, and III respectively showing that the lung is formed of normally looking alveoli with patent lumens (A) and thin interalveolar septa (thin arrows). Notice the alveolar sacs (S), and the intact bronchiole (B). **c** Group II showing thick interalveolar septa (arrow) with inflammatory cell infiltration (circle). Some alveoli appeared narrow (A) and others dilated (d), while others showing intraluminal debris (red circle). The bronchioles (b) appeared with a wide lumen surrounded by inflammatory cells (circle). The bronchial epithelium appears with focal disruption. Notice the dilated congested blood vessel (BV) surrounded by an edematous area (o). **e** Group IV showing lung tissue with thin interalveolar septa (blue arrow) but still thick septa (black arrow) seen. Notice the less inflammatory cell infiltration (circle). H&E × 100

**Fig. 2:**
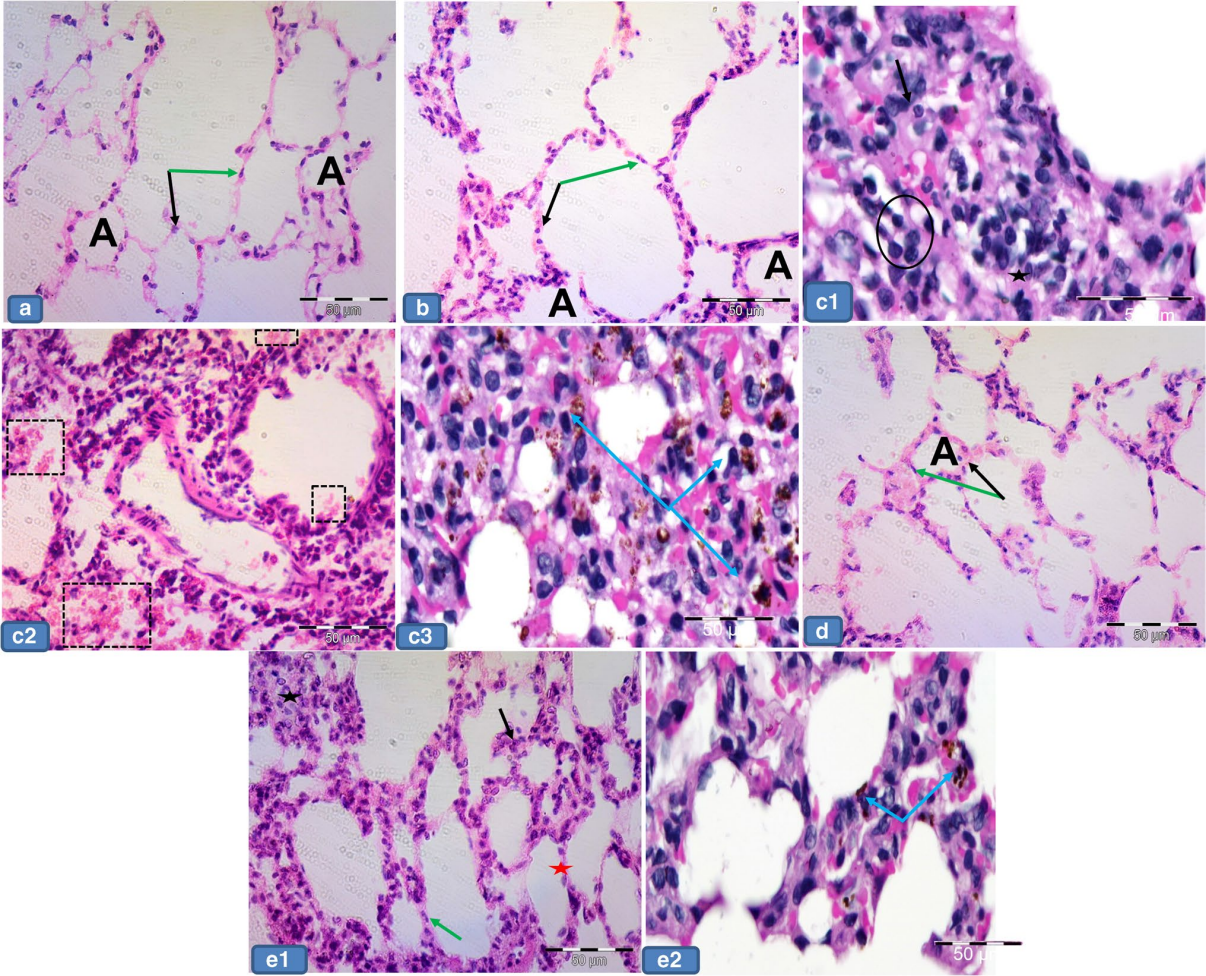
Representative Photomicrographs of lung sections of adult rats. **a**, **b**, and **d** Group Ia, Ib, and III respectively showing alveoli (A) lined by pneumocyte type 1 with their flat nuclei (green arrows) and few rounded type II pneumocytes (black arrows) with their central round vesicular nuclei. **c** Group II showing **c1** thick interalveolar septa (black star). Notice the numerous pneumocyte type II (black arrow) aggregated in many areas (encircled). **c2** Intra-alveolar and intra-bronchial blood cells are noticed (square). **c3** Showing macrophage with brownish hemosiderin granules settles in the inter-alveolar septa (blue arrows). **e** Group IV showing **e1** Thin interalveolar septa (red star) but still thick septa are observed (black star). Notice the less numerous pneumocyte type II (black arrow). **e2 **Showing less numerous macrophages in inter-alveolar septa (blue arrows). H&E × 400

**Fig. 3:**
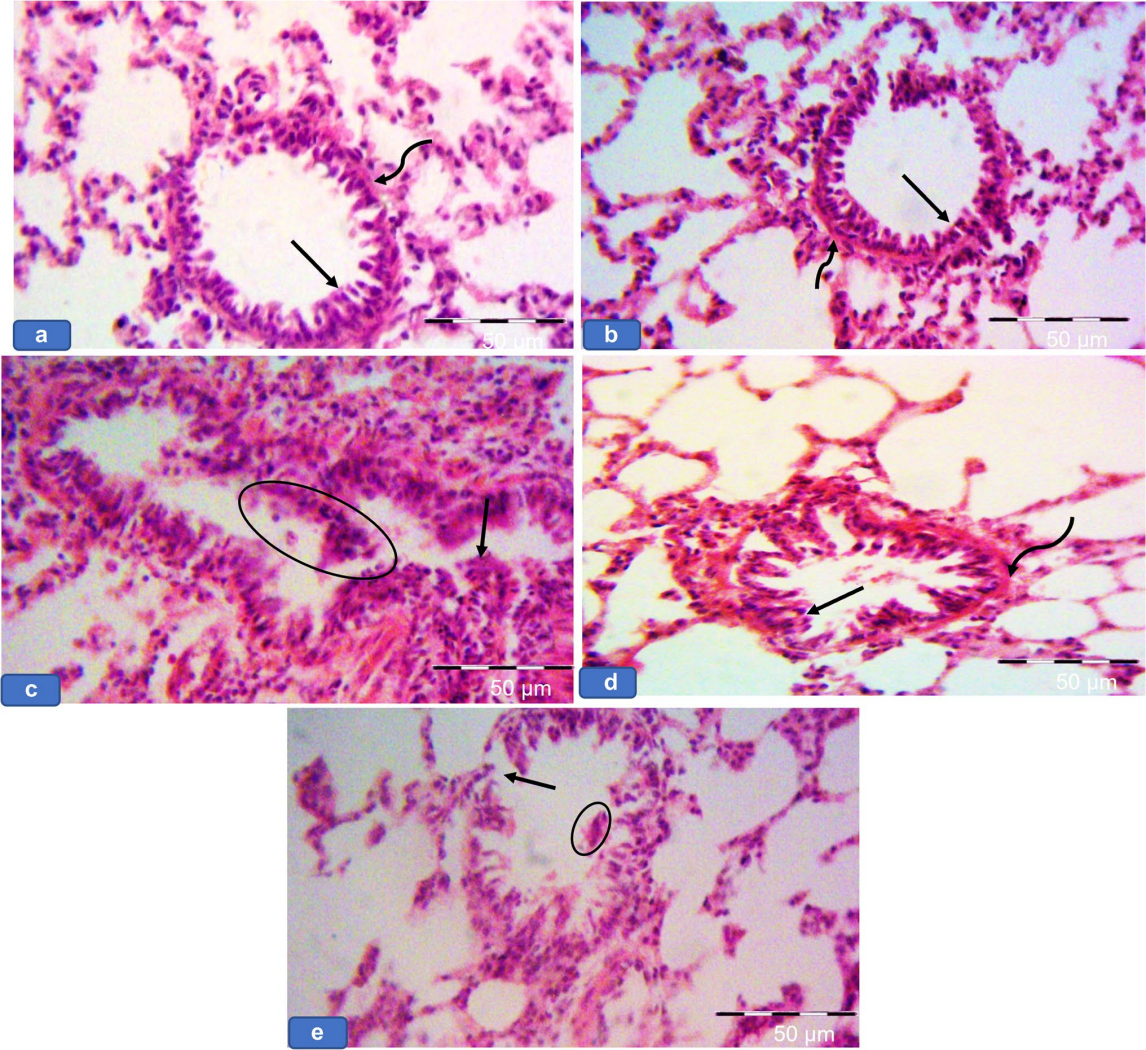
Representative Photomicrographs of lung sections of adult rats: **a**, **b**, **d** Group Ia, Ib, and III, respectively showing bronchioles lined by ciliated columnar epithelium with few goblet cells (arrows) surrounded by a thin muscle cell layer (curved arrows). **c **Group II showing dilated bronchioles with disturbed epithelium lining. Notice the intraluminal bronchial debris (circle). **e** Group IV showing bronchioles more or less normal except for focal disturbed epithelial lining (black arrow) with few intraluminal cellular debris (circle) (H&E × 400)

Lung sections from groups Ia, Ib, and III showed normal histological features of the lung. The alveoli were observed with normal alveolar lumina with thin interalveolar septa. Alveolar sacs, alveolar duct, and the intact bronchiole were seen (Fig. 1a, b, d). The alveoli were lined by type I pneumocytes with flat nuclei and few rounded type II pneumocytes with central rounded vesicular nuclei (Figs. 2a, b, d). The bronchioles were lined by simple columnar ciliated epithelium with few goblet cells surrounded by thin muscle cell layers (Figs. 3a, b, d).

In contrast, group II showed thick interalveolar septa with inflammatory cells. Some alveoli appeared narrow; others appeared dilated while others appeared with intraluminal debris. More numerous aggregated pneumocyte type II were seen in many areas. Dilated congested blood vessel surrounded by perivascular inflammation was seen. Intra-alveolar and intrabronchial blood cells were also noticed. Additionally, macrophages with brownish hemosiderin granules settled in interalveolar septa were noticed among the sections (Figs. 1c1, c2, 3c1, c2, and c3).

The bronchioles appeared with a wide lumen and disturbed epithelium surrounded by peri bronchial inflammatory cell infiltration and most probably edematous areas. Intraluminal bronchial debris was also seen among the sections (Fig. 3c).

However, group IV exhibited thin interalveolar septa while others displayed thick septa (Fig. 1e). Less numerous pneumocyte type II were observed if compared to group II. Less surrounding inflammatory cell infiltration was noticed (Fig. 2e1). Additionally, less numerous macrophages settled in interalveolar septa were noticed if compared to group II (Fig. 2e2). The bronchioles were noticed more or less normal except for focal disturbed epithelial lining with few intraluminal cellular debris (Fig. 3e).

### Anti-cleaved caspase immunostaining results

**Fig. 4:**
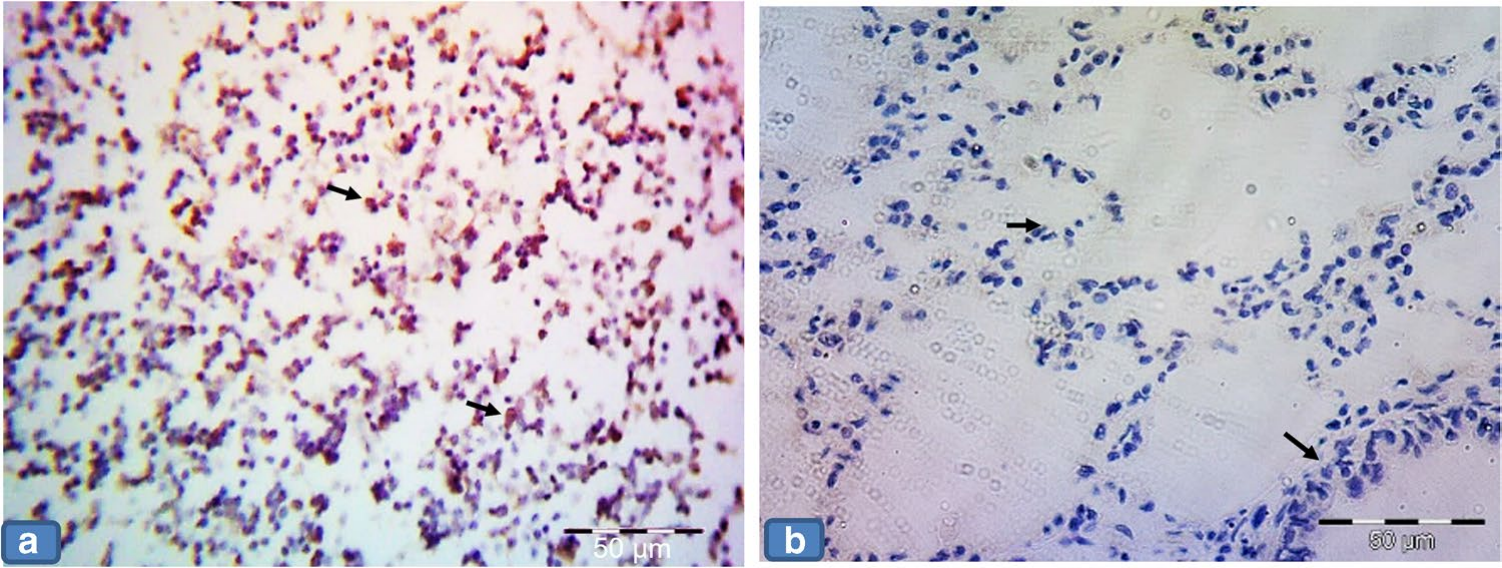
Representative photomicrographs of **a** Positive control slide for cleaved anti-caspase in rat tonsillar tissue showing cytoplasmatic and nuclear expression in most properly macrophage-like cells (arrows). **b** Negative control slide showing no reaction in alveolar nor bronchial cells (arrows). × 400

**Fig. 5:**
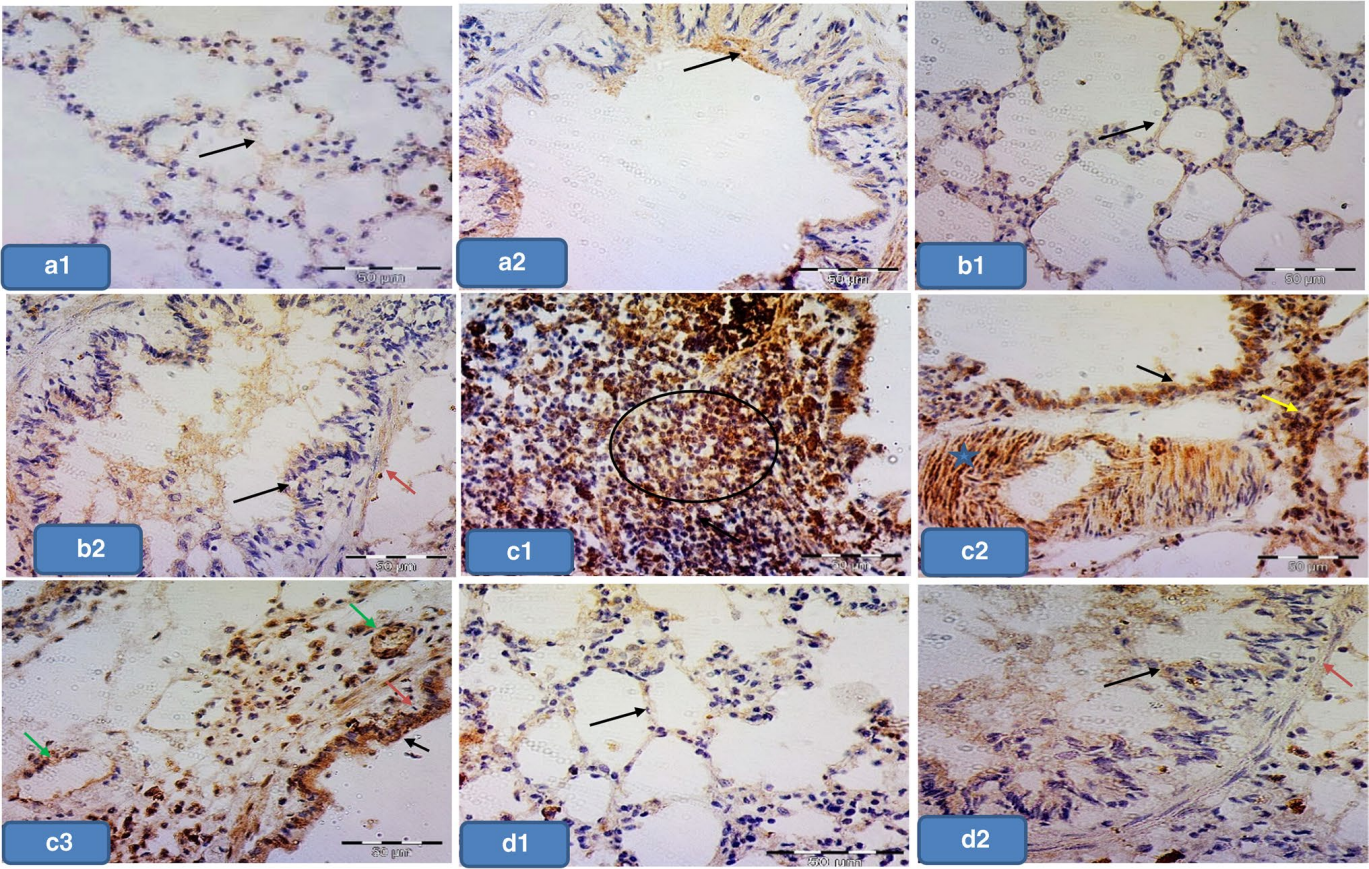
Representative photomicrographs of lung sections of adult rats from all groups stained with cleaved anti-caspase immunostaining: **a1** and **a2**, **b1** and **b2**, **d1** and **d2** Group Ia, Ib, and III respectively showing faint immunoreaction in both alveolar and bronchial epithelium (black arrows). Also, notice the immunoreactivity in the smooth muscle layer of bronchioles (red arrows). **c1**, **c2**, **c3** Group II showing positive cytoplasmic immunoreactivity in the pneumocyte cells (yellow arrow), in the lining epithelium (black arrow), and smooth muscle layer of bronchioles (red arrow). Notice also the positive staining in the arterial wall (star), capillary endothelium (green arrow), and in the inflammatory cells (circle) within the thickened interalveolar septa. **e1** and **e2** Group IV showing less positive immunoreactivity in the pneumocyte cells (yellow arrow), in the lining epithelium (black arrow), and smooth muscle layer of bronchioles (red arrow). Notice the less stained inflammatory cells (circle). Cleaved anti-caspase counterstained with H&E × 400

The positive control slides for anti-caspase showed cytoplasmatic and nuclear expression in most properly macrophage-like cells (Fig. 4a). The negative control slides showed no reaction in the alveolar nor bronchial epithelial lining cells (Fig. 4b), while groups Ia, Ib, and III displayed faint immunoreaction in the epithelial lining of both alveoli and bronchioles. The immunostaining in the smooth muscle layer of bronchioles was also noticed (Fig. 5a1 and a2, b1 and b2, d1 and d2), while group II showed positive cytoplasmic immunoreaction in pneumocyte cells. The epithelium lining and smooth muscle layer of bronchioles also showed this cytoplasmic expression. Positive staining in the arterial wall and endothelium lining of blood capillaries was also observed. There was positive immunoreactivity within the inflammatory cells lying in the thickened interalveolar septa (Fig. 5c1 and c2). In contrast, group IV revealed less positive cytoplasmic immunoreactivity in the pneumocyte cells if compared to group II. There was also less positive cytoplasmic immunoreactivity in the inflammatory cells settled in the alveolar lumen (Fig. 5e1 and e2).

### BAL fluid result

**Fig. 6:**
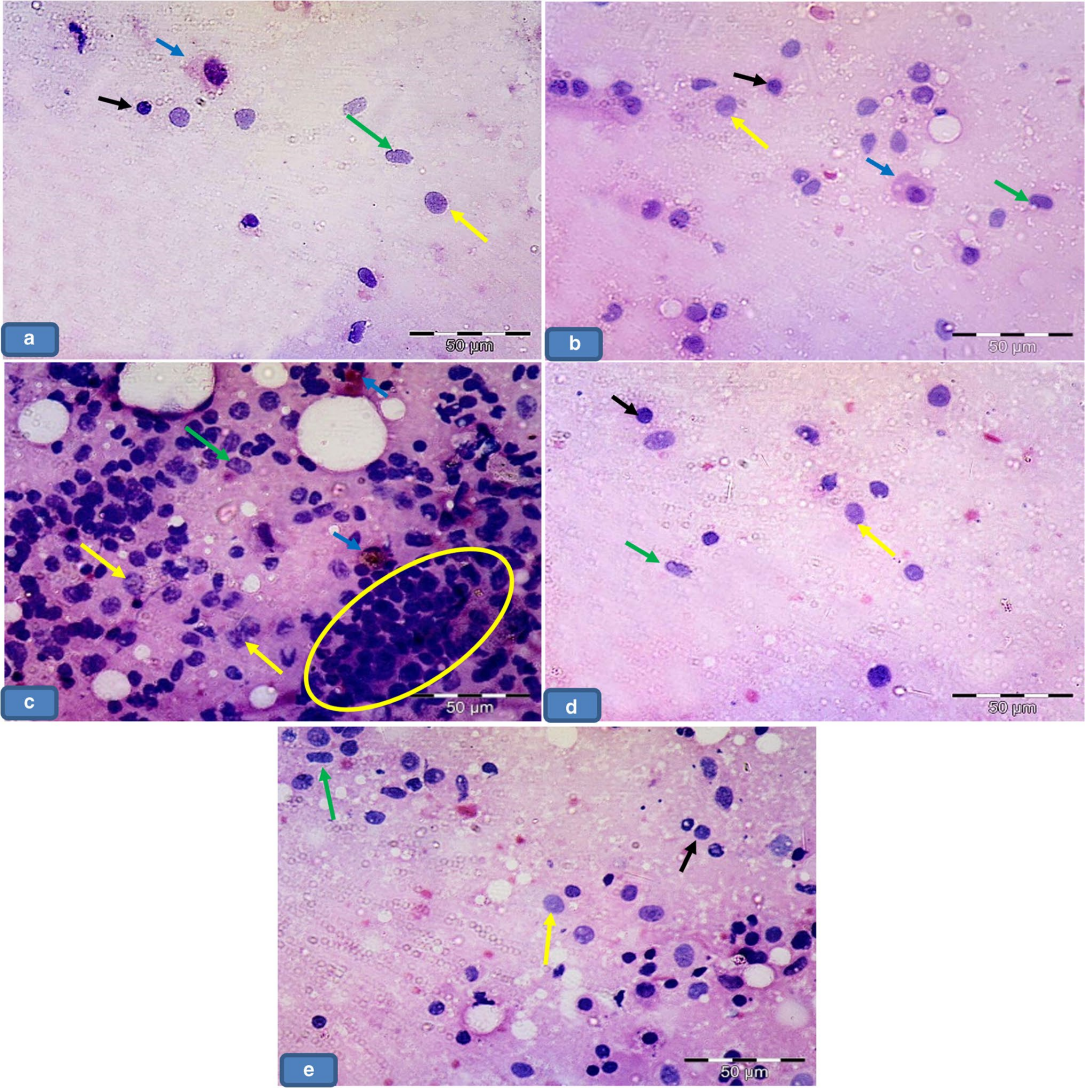
Microscopic aspect of slides with BAL fluid after centrifugation showing **a**, **b**, and **d** Group Ia, Ib, and III respectively showing pneumocyte type 1 (green arrows), lymphocytes (black arrows), and macrophages (blue arrows). **c** Group II showing pneumocyte type 1 (green arrows), numerous pneumocyte type II (yellow arrow), numerous lymphocytes (circle), and macrophages (blue arrows) laden with cytoplasmic brownish hemosiderin granules. **e** Group IV showing less numerous pneumocyte type 1 (green arrows), pneumocyte type II (yellow arrow), and lymphocytes (black arrows). BAL fluid × 400

The microscopic study of slides with BAL fluid after centrifugation in groups Ia, Ib, and III showed few pneumocyte type 1, lymphocytes, and macrophages (Fig. 6a, b, d), while group II showed pneumocyte type 1, numerous pneumocyte type II, numerous lymphocytes, and macrophages laden with cytoplasmic brownish hemosiderin granules (Fig. 6c). In contrast group IV, exhibited less numerous pneumocyte type 1, pneumocyte type II, and lymphocytes compared to group II (Fig. 6e).

### Semi-thin section results

**Fig. 7:**
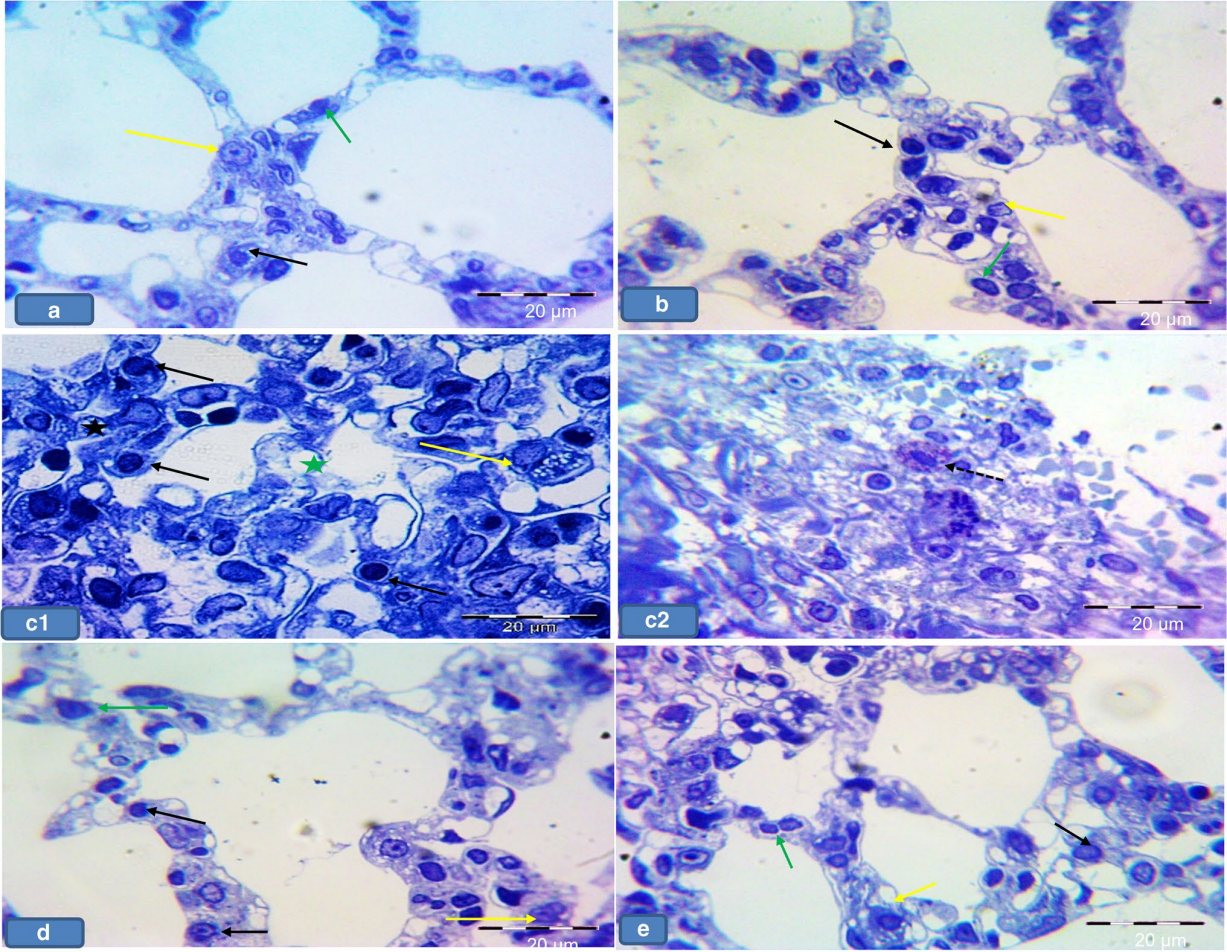
Representative photomicrographs of lung sections of adult rats from all groups showing **a**, **b**, and **d** Group Ia, Ib, and III, respectively showing alveoli lined with pneumocyte type I (green arrows) with flatted nuclei and pneumocyte type II (black arrows) with rounded nuclei. Notice macrophages in the thin inter-alveolar septa (yellow arrows). **c** Group II showing **c1** thick interalveolar septa (star), multiple pneumocyte type II (black arrow) and multiple large-sized macrophages (yellow arrows). Notice the necrotic area (green star). **c2** Showing a purple-stained mast cell (dotted arrow) with cytoplasmic granules. **e** Group IV showing less numerous macrophages (yellow arrows) and less numerous pneumocyte type II (black arrow). Toluidine blue × 1000

Groups Ia, Ib, and III showed pneumocyte type I with flatted nuclei and pneumocyte type II with rounded nuclei lining the alveoli. The interalveolar macrophages with irregular nuclei were noticed (Fig. 7a, b, d), while group II showed thick interalveolar septa with numerous pneumocyte type II and multiple large-sized macrophages. Focal necrotic areas were also detected. Large-sized mast cells-stained purple with cytoplasmic granules were seen in this group (Fig. 7c1 and c2). Meanwhile, group IV revealed less numerous macrophages with less numerous pneumocyte type II if compared with group II (Fig. 7e).

### Electron microscopic examination

**Fig. 8:**
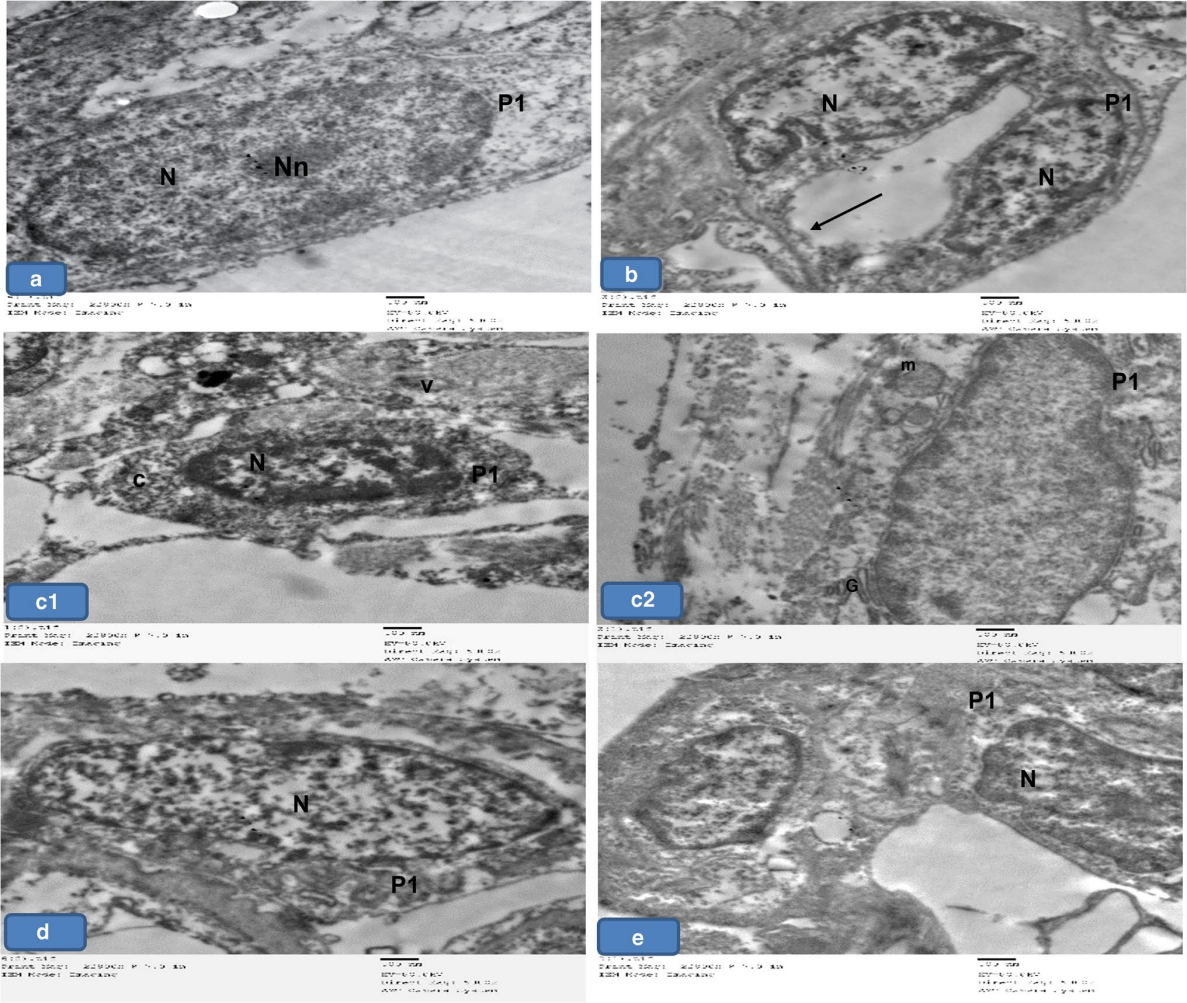
Representative electron micrographs of lung tissue of adult rats from all groups showing **a**, **b**, and **d** Group Ia, Ib, and III respectively showing normal type 1 pneumocyte (P1) with flattened euchromatic nuclei (N) and prominent nucleoli (NU). Notice the thin inter-alveolar septum (arrow). **c** Group II showing pneumocyte type 1 (P1) with heterochromatic nuclei and dense cytoplasm (c) surrounded by vacuolated area (v). Notice the dilated cisterns of rough endoplasmic reticulum (rER), Golgi apparatus (g), and dilated distorted mitochondria (m). **e** Group IV showing nearly normal pneumocyte I (P1). × 5000

Lung sections of groups Ia, Ib, and III showed that the alveoli were lined with type I pneumocytes with flat euchromatic nuclei and prominent nucleoli separated by thin interalveolar septate (Fig. 8a, b, d), while group II showed pneumocyte type 1 with heterochromatic nuclei with dense cytoplasm surrounded by vacuolated areas. Their cytoplasm appeared with dilated cisterns of rough endoplasmic reticulum, Golgi apparatus, and dilated distorted mitochondria (Fig. 8c1 and c2). Furthermore, pneumocyte type I in group IV appeared more or less normal (Fig. 8e).

**Fig. 9:**
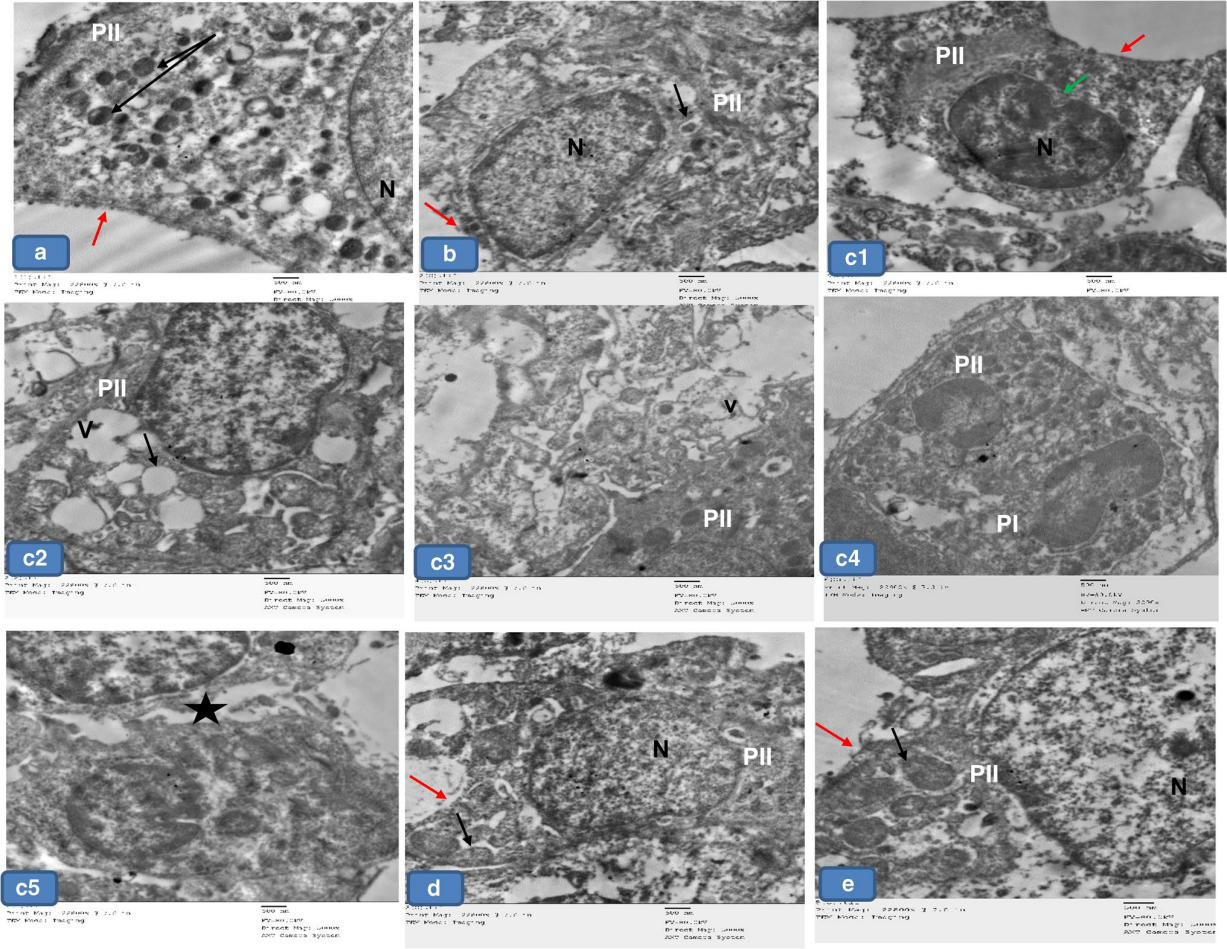
Representative electron micrographs of lung tissue of adult rats from all groups showing **a**, **b**, and **d** Group Ia, Ib, and III, respectively showing normal type II pneumocyte (PII) with large rounded euchromatic nuclei (N) and apical microvilli (red arrows). Notice the lamellar bodies (black arrows). **c** Group II showing pneumocyte type II (PII) with shrunken indented heterochromatic nucleus (green arrow) with dense vacuolated cytoplasm (v). Notice the empty lamellar bodies (black arrow) and cytoplasmic vacuolations (V). Loss of apical microvilli is seen (red arrow). The cells are widely separated (star). The degenerated Pneumocyte type II (PII) is surrounded by degenerated pneumocyte type I (PI). **e** Group IV showing pneumocyte II (PII) with nearly normal structure except the loss of apical microvilli (red arrow). × 5000

**Fig. 10:**
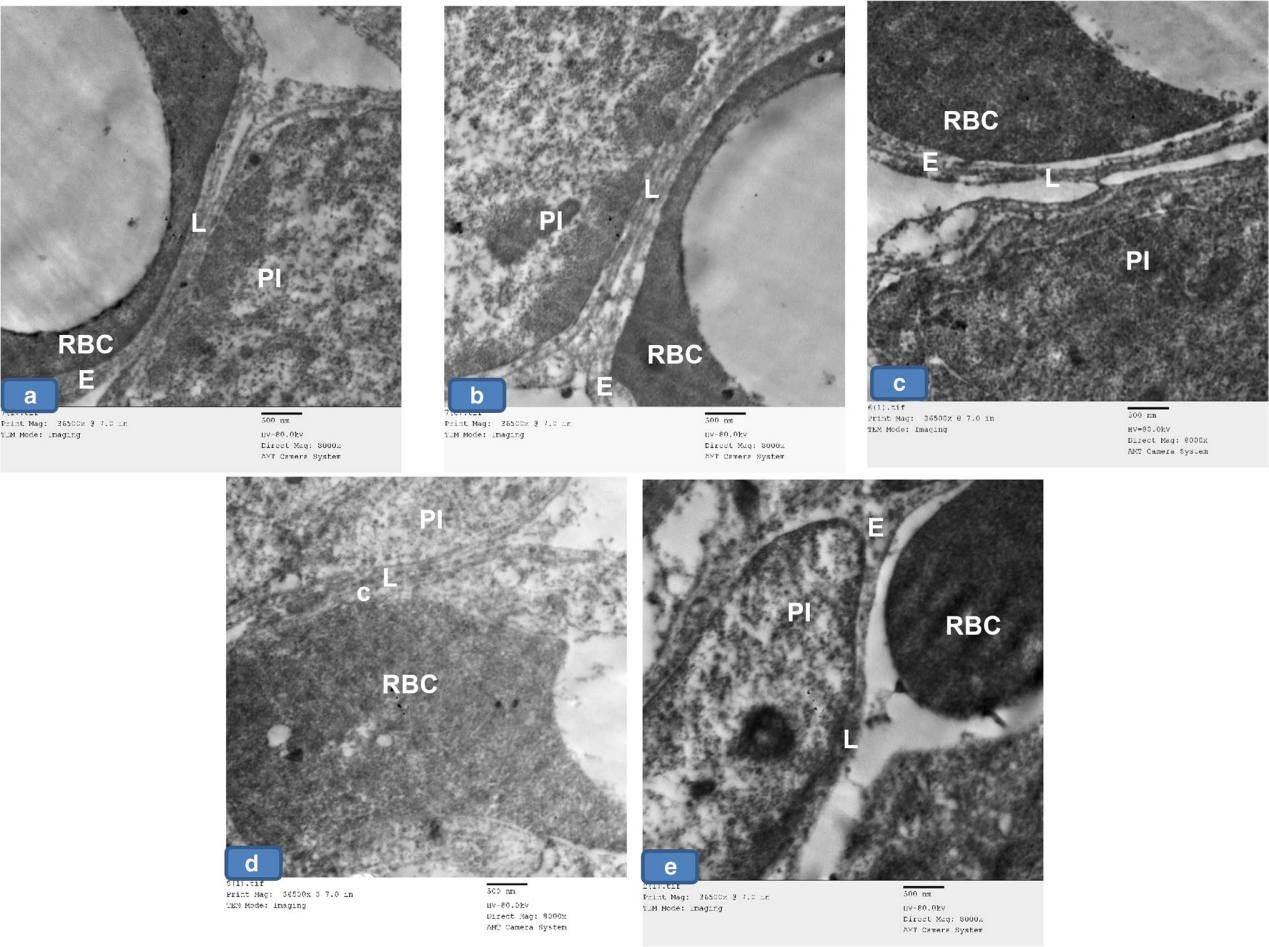
Representative electron micrographs of lung sections of adult rats from all groups showing **a**, **b**, and **d** Group Ia, Ib, and III, respectively showing the blood air barrier with pneumocyte type I (PI), fused basal lamina (L), and cytoplasm (E) of capillary endothelial cells. **c** Group II showing the blood- air barrier having swollen irregular cytoplasm of pneumocyte type I (PI), thick irregular fused separated basal lamina (L), and cytoplasm (E) of capillary endothelial cells. **e** Group IV showing more or less normal air–blood barrier. × 5000

Type II alveolar cells were seen lined the alveoli in groups Ia, Ib, and III. They appeared with large euchromatic rounded nuclei. The cytoplasm showed multiple lamellar bodies (Fig. 10a, b, d). In group II, pneumocyte type II appeared with indented heterochromatic nuclei, and dense vacuolated cytoplasm surrounded by vacuolated areas, whereas, other cells appeared shrunken. The empty lamellar bodies and cytoplasmic vacuolations were seen among the sections. Loss of apical microvilli was also noticed. These cells were noticed widely separated. The degenerated pneumocyte type II were seen surrounded by degenerated pneumocyte type I (Fig. 9; c1, c2, c3, c4, c5). Meanwhile, group IV showed pneumocyte type II with nearly normal structure except for the loss of their apical microvilli (Fig. 10e).

Regarding the blood-air barrier, groups I and III showed pneumocyte type I, fused basal lamina, and cytoplasm of capillary endothelial cells (Fig. 10a, b, d), while group II showed blood air barrier with swollen irregular cytoplasm of pneumocyte type I, thick irregular fused separated basal lamina, and cytoplasm of capillary endothelial cells (Fig. 10c). Furthermore, group IV showed the blood-air barrier approached normal structure (Fig. 10e).

### Morphometric and statistical results

There was a significant increase in both the mean surface area fraction of cleaved caspase 3 and the mean number of pneumocyte type II in group II if compared with groups Ia, Ib, and III. While there was a significant decrease in these parameters in group IV if compared with group II (Histogram 1). Histopathological severity of lung injury was significantly reduced in group IV if compared to group II. Scoring results of inflammation and destruction of lung tissue are shown in Table 5.

**Histogram 1:**
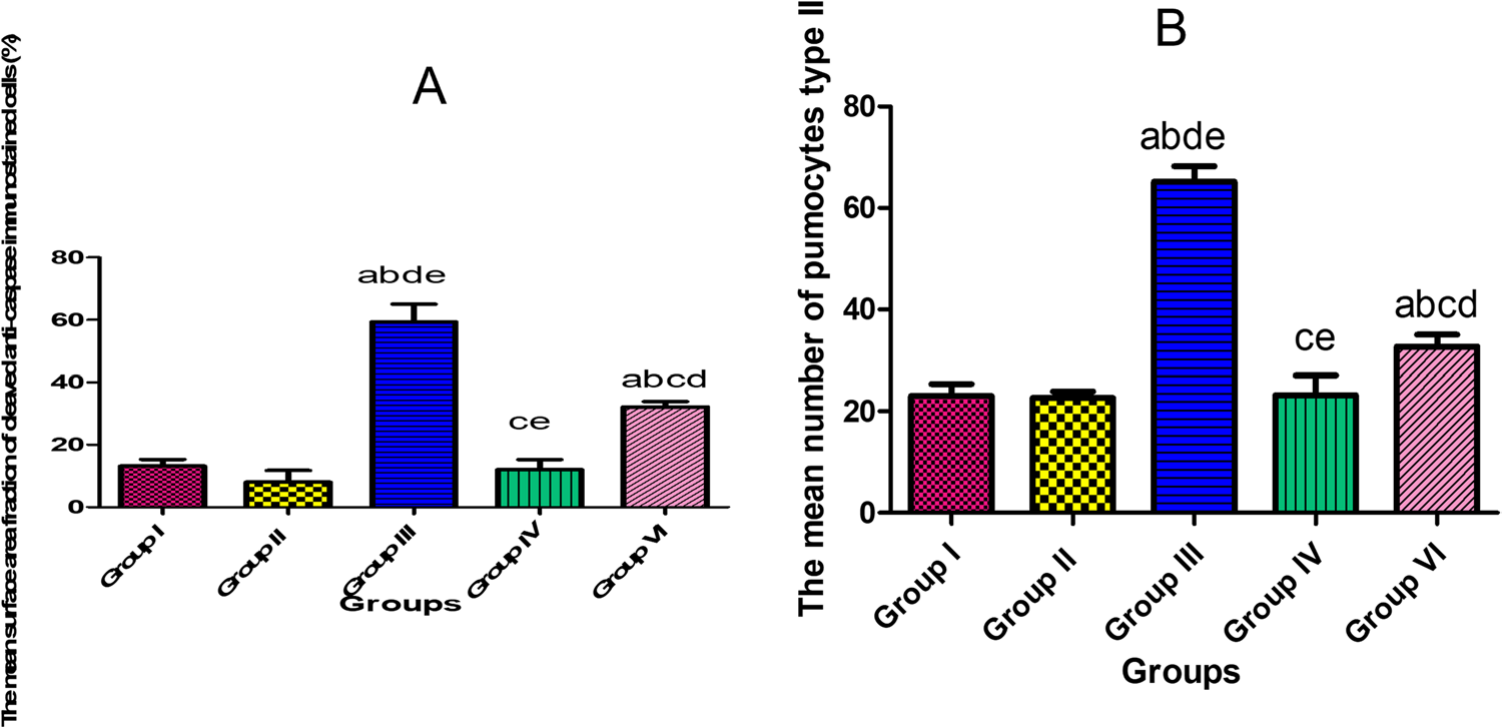
**A** The mean surface area fraction of cleaved anti-caspase 3 in all studied groups. **B** The mean number of pneumocyte type II in all studied groups. Results represent the mean SD (*n* = 6). a—Significant (*P* < 0.05) difference from group I, b—significant (*P* < 0.05) difference from group II group, c—significant (*P* < 0.05) difference from group III, d—significant (*P* < 0.05) difference from group IV, e—significant (*P* < 0.05) difference from group V

**Table 5:** Scoring results of inflammation and destruction of lung tissue in the different experimental groups

Groups	Control groups		Group II	Group III	Group IV
Parameters	Ia	Ib			
Scoring results of inflammation and destruction)	0.0 ± 0.0	0.0 ± 0.0	7.000 ± 0.8944^**ab**^	0.0 ± 0.0^**c**^	4.000 ± 0.6325^abcd^

Results represent the mean ± S.D. ^a^Significant difference from control group Ia, ^b^significant difference from control group Ib, ^c^significant difference from group II; ^d^significant difference from group III, *P* < 0.05. *MDA*, malondialdehyde; *Nrf2*, nuclear factor erythroid 2-related factor 2; *TNF-α*, tumor necrosis factor-alpha (*n* = 8)

## Discussion

Clinically, acute lung tissue injury is a serious complicated condition responsible for the majority of acute respiratory failure in seriously ill patients worldwide. Its treatment is still largely supportive, creating an ongoing need for updated research (Fan et al. 2022). The lung tissue contains low levels of the enzyme and therefore is more susceptible to bleomycin-induced tissue injury (Pandey et al. 2021). The current work aimed to assess the possible protective effects and the mechanism of protection of 3,3′-methylenebis-(1-ethyl-4-hydroxyquinolin-2(1*H*)-one) on BLM-induced lung injury in addition to the effect and underlying mechanisms of nuclear factor-erythroid-related factor 2 pathway against this injury.

BLM which is an antibiotic secreted by the bacterium named *Streptomyces* *Verticillus* (Chen et al. 2020), is one of the first described chemotherapeutic agents that has been used for cancer treatment. In the current study. Intratracheal route was used which is not applied in clinical practice just to ensure induction of lung injury. Intratracheal route of administration is the commonest route to induce and mimic idiopathic lung injury in many studies with fewer systemic manifestations (Aokage et al. 2021).

BLM group revealed various changes such as inter-alveolar septal thickening which may be due to severe mononuclear cellular infiltration, numerous pneumocyte type II, and alveolar exudates due to secretions and remnants of dead cells as pneumocytes and macrophages. These histological changes coincide with other studies which used BLM on lung tissue (Knudsen et al. 2018). Additionally, these results were in line with a previous histopathological study which reported that BLM can promote acute cellular inflammation in lung tissue as demonstrated by a strong influx of inflammatory cells infiltration e.g. macrophages and activation of fibroblasts (Skurikhin et al. 2015).

Malondialdehyde (MDA) was reported as a marker for oxidative stress-induced lipid peroxidation as documented previously (Gaweł et al. 2004). BLM group displayed a significant increase in lung tissue MDA concentration. It was accompanied by parenchymal inflammatory cell infiltration with abundant macrophages in bronco alveolar lavage fluid. It was in accordance with a previous work that stated that there was an imbalance between both pro and antioxidant defense enzymes with BLM treatment. It was documented that oxidative stress promotes both the recruitment and stimulation of resident neutrophil and macrophage cells, resulting in tissue injury. Exaggerated neutrophilic cell infiltration is the main cause of the injury of the alveolar-capillary barrier (Saber et al. 2018) (Kang et al. 2020).

The previous findings of BLM-induced oxidative stress cytotoxicity further explained that the BLM may lead to single and double-stranded breaks in DNA and rapid DNA fragmentation. In addition to that, BLM is inactivated by bleomycin hydrolase, an enzyme found in most tissues except for the lungs and skin so it can affect the lung if administered parenterally or direct intratracheally which was more effective in inducing lung injury. Furthermore, the chelation of iron ions with oxygen resulted in the production of DNA-cleaving of both superoxide and hydroxide free radicals that resulted in BLM lung toxicity, and pulmonary fibrosis (Elgendy et al. 2021).

On the other hand, the levels of antioxidant enzymes (GSH and SOD) were used to assay the oxidative stress and the synthesis of reactive oxygen species. It was in line with a previous study that used BLM (Badawi 2022).

Interleukin-1 (IL-10) is secreted by both T helper lymphocytes and mononuclear macrophages. It exerts an anti-inflammatory effect via T cells. IL-10 regains the balance between anti-inflammatory and inflammatory response via suppression of the body’s inflammatory response (Qin and Qiu 2019). It was noticed a significant increase in this parameter in group II if compared to groups I and III.

Nuclear factor erythroid 2-related factor (Nrf2) is a ubiquitously expressed reductase-sensitive transcription factor that is maintained in a dormant state by its interaction with an inhibitory protein, Kelch ECH protein 1 (Keap-1). In response to an increase in the free radicals, the Keap protein is oxidized or covalently modified and releases Nrf2, which then enters the nuclei and bonded to the antioxidant response element (ARE), thus stimulating transcription of a group of antioxidant genes e.g. superoxide dismutase (SOD), catalase, heme oxygenase 1 (HO-1), and NAD (P) H: quinone oxidoreductase 1 (NQO1) (Sharma et al. 2017). Furthermore, Nrf2 had reported anti-inflammatory properties driven by its ability to negatively control nuclear factor-kappaB (NF-kB), the transcription factor central to the inflammatory response (Audousset et al. 2021). BLM group showed a significantly decreased in pulmonary Nrf2 concentration.

Mast cells were detected between lung tissue sections in the BLM-treated group. Mast cells play a main role in acute lung tissue diseases. Their activation lead to degranulation and release of inflammatory cell mediators e.g. histamine, cytokines, proteases, chymase, and tryptase (Huang et al. 2012). Histamine and tryptase activate inflammatory cell infiltration and promote cytokine generation as proved in the current work by significantly increased pulmonary TNF-α concentration as detected previously (Peritore et al. 2021).

There was histological evidence of lung injury in the BLM group as thickening of alveolar walls, swelling, fragmentation of alveolar epithelial cells, and cellular infiltrates. These changes could be due to alterations of the alveolar-capillary barrier (Steffen et al. 2017) in addition to inflammatory responses as evidenced in the present study by increasing the inflammatory markers as TNF-α.

More numerous macrophages containing brownish cytoplasmic pigment most properly resulting from red blood cell lysis. Macrophages aggregated in lung tissue early just after damage induced by lung toxicants were noticed to be stimulated toward a pro-inflammatory M1 phenotype, as evidenced by morphologic changes and increased size and vacuolization, and concentration of lung TNF-α, a prototypical mark of pro-inflammatory/cytotoxic macrophages (Malaviya et al. 2017).

As regards the alveolar lining cells in the BLM group, the current study reported that there were several morphological alterations in both types I and II alveolar epithelial cells revealed by light and electron microscopy as a marked decrease in type I alveolar cells, appearance of few vacuolated cells and many cuboidal cells which were detected by electron microscopy to be type II alveolar cells. So, type II alveolar cells were the predominant cells lining the alveoli. Similar changes in the epithelial lining were previously reported by another study (Mahdi and Ibrahim 2015) that attributed these findings to the destruction of type II alveolar cells with subsequent replacement by the proliferating type II alveolar cells. They also reported that as type I cells represent the majority of the alveolar lining cells, so they are more susceptible to injury with a subsequent increase in pulmonary epithelial permeability in BLM-induced interstitial pneumonitis.

In addition, the data of the present study revealed that many type II alveolar cells showed some degenerative changes as empty lamellar bodies and loss of microvilli in BLM-treated group. These changes were suggested to be caused by the disturbed biochemical processes that involved the membranous components of the cytoplasm which resulted in an imbalance between the distribution of fluids and electrolytes (Nakama et al. 1998). The disturbance in alveolar type II epithelium is matching with a significant decrease in plasma level of surfactant protein-A (SP-A) in the BLM-treated group. This was in accordance with Watson et al. (2021) who suggested that injury of alveolar epithelial cells in acute lung inflammation resulted in impaired synthesis, secretion, and composition of surfactant proteins and added that lung SP-A plays the main role in controlling innate immunity in the lungs as it has the ability to suppress the bacterial growth and stimulate macrophage phagocytosis.

In this research paper, the caspase-3 immuno-expression was significantly increased in the BLM group compared with control subgroups. This result agrees with a previous histopathological study (Elgendy et al. 2021; Kim et al. 2021). Some proinflammatory cytokines such as IL-1β, TNF-α, and IFN-γ induce cell death (apoptosis) in various cell types. Apoptosis is executed by caspase-3 and -7 (Karki et al. 2021).

In the current work, in group IV when we used 3,3′-methylenebis(1-ethyl-4-hydroxyquinolin-2(1*H*)-one) with BLM as another modality line of treatment of BLM-induced lung injury. H & E sections showed nearly normal architecture with apparently thin interalveolar septa, few cellular infiltrations, and less congested blood vessels. Alveoli appeared lined by pneumocyte type I and II without remarkable proliferation or any remnants of dead cells. The septa between alveoli appeared thin. Lamellar bodies with thin fine lamellae were observed but still, some lamellar bodies were empty. Additionally, the mean surface area fraction of caspase 3 and the number of pneumocytes II were decreased in this group if compared with group II. In addition, the biochemical results showed that of the pretreatment the BLM-treated rats by 3,3′-methylenebis(1-ethyl-4-hydroxyquinolin-2(1*H*)-one) significantly increased the plasma level of SP-A with decreased of pulmonary MAD and TNF α concentrations.

## Conclusion

It could be concluded that BLM has profound cytotoxic effects on lung tissue. 3,3′-methylenebis(1-ethyl-4-hydroxyquinolin-2(1*H*)-one) as a natural antioxidant with anti-inflammatory and anti-apoptotic effects, improved BLM-induced lung injury except for minimal biochemical and histological changes.

## Recommendation

A similar study on human subjects is recommended to determine whether these results could be applied to humans or not. Furthermore, long-term exposure to BLM is recommended.

## Data Availability

The data that support the findings of this study are available from the corresponding author upon reasonable request.

## Abbreviations

BLM: Bleomycin
ALI: Acute lung injury
IPF: Idiopathic pulmonary fibrosis
Nrf2: Nuclear factor-erythroid-related factor 2
Gpx1: Glutathione peroxidase 1
MDA: Malondialdehyde
GSH: Reduced glutathione
TNF-α: Tumor necrosis factor-alpha
IL-10: Interleukin 10
FBAL: Fractional bronchoalveolar lavage
H&E: Hematoxylin and eosin
SP-A: Surfactant protein A
HO-1: Heme oxygenase 1
